# Case Report: Ultrasound guided puncture for type 2 diabetes mellitus combined with psoas abscess—a report of two cases

**DOI:** 10.3389/fmed.2026.1773238

**Published:** 2026-04-08

**Authors:** Liang Chen, Yiqiang Ding, Yun Liu, Qi Xie, Jiabin Hu, Mingxing Wang, Xiaoqin Zeng, Deming Zou

**Affiliations:** 1 Department of Pulmonary and Critical Care Medicine, Xingguo People’s Hospital, Xingguo Hospital Affiliated to Gannan Medical University, Ganzhou, China; 2 Department of Pharmacy, Xingguo People’s Hospital, Xingguo Hospital Affiliated to Gannan Medical University, Ganzhou, China; 3 Department of Oncology, Xingguo People’s Hospital, Xingguo Hospital Affiliated to Gannan Medical University, Ganzhou, China; 4 Department of Nephrology, Xingguo People’s Hospital, Xingguo Hospital Affiliated to Gannan Medical University, Ganzhou, China; 5 Department of Infectious Diseases, Xingguo People’s Hospital, Xingguo Hospital Affiliated to Gannan Medical University, Ganzhou, China

**Keywords:** metagenomic next-generation sequencing, percutaneous puncture drainage, psoas abscess, type 2 diabetes mellitus, ultrasound intervention

## Abstract

**Background:**

Psoas abscess (PA) is a rare infectious disease, with type 2 diabetes mellitus (T2DM) serving as a significant risk factor. The combination of metagenomic next-generation sequencing (mNGS) and ultrasound offers innovative approaches for the rapid and precise treatment of PA.

**Case presentation:**

Case 1: A 77-year-old woman presented with lumbar pain was initially misdiagnosed with lumbar disc herniation based on CT scan. Subsequent CT scan and ultrasound-guided puncture confirmed a left lumbar PA. mNGS detected the presence of *Streptococcus agalactiae*, which was negative on conventional culture. The patient was successfully treated with vancomycin for 5 weeks, with no recurrence at 3-year follow-up. Case 2: A 56-year-old woman with a 10-year history of T2DM presented with poor appetite and fatigue. CT imaging identified a left lumbar PA along with perirenal infection. Pus from ultrasound-guided puncture for conventional culture and mNGS detected the presence of *Staphylococcus aureus*. Treatment with oxacillin and vancomycin led to clinical resolution. The follow-up CT scan in 2024 indicated complete resorption of the lesion.

**Conclusion:**

mNGS combined with ultrasound-guided puncture overcomes conventional culture limitations. This approach suggests clinical feasibility.

## Introduction

Psoas abscess (PA) is a rare infectious disease that is frequently overlooked or misdiagnosed due to its insidious onset and the lack of specificity in its clinical manifestations (1). Diabetes mellitus, a significant risk factor for PA, can elevate the risk of infection through mechanisms such as immune function suppression and impaired neutrophil activity (2). Accurate pathogenic diagnosis is crucial for the treatment of PA (3). Traditional culture methods exhibit low positivity rates; in contrast, metagenomic next-generation sequencing (mNGS) offers rapid and unbiased results and has been widely utilized in infectious disease diagnostics for pathogen screening (4). This paper analyzes the diagnosis and treatment of two cases of diabetes mellitus combined with PA, aiming to explore the clinical applicability of ultrasound intervention combined with mNGS in the management of PA.

## Case presentation

### Case 1

**Figure 1:**
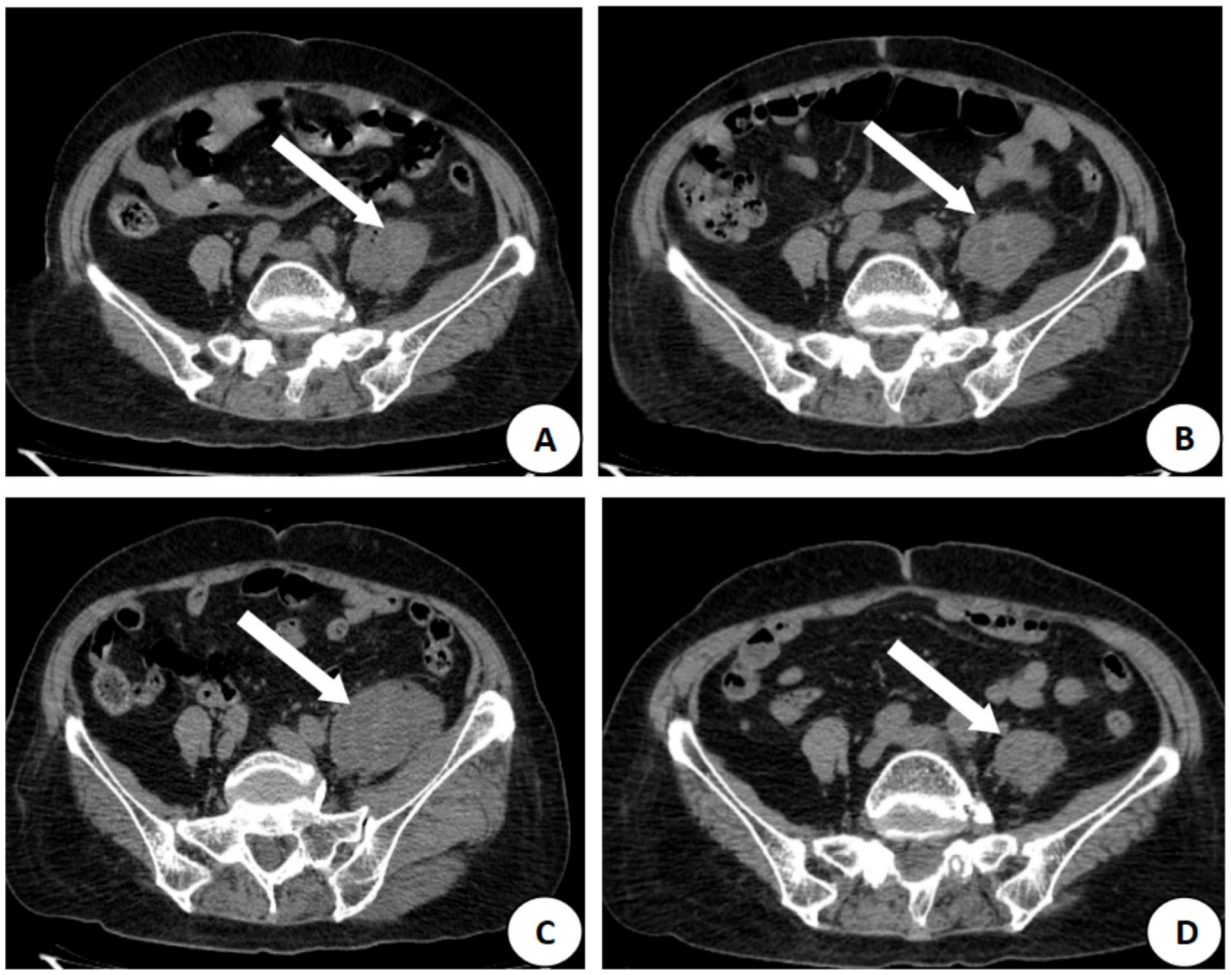
CT image of the psoas major muscle of the patient in Case 1. **(A)** Thickening of the left psoas major muscle, abscess formation, and arrows show punctate pneumatization on March 19, 2021; **(B)** Increase in the volume of the abscess, and disappearance of the pneumatization on April 1, 2021; **(C)** Further enlargement of the abscess, and the arrows show the maximal diameter of up to 3.2 cm on April 10, 2021; **(D)** Absorption of the abscess, and only mild inflammation remains, arrows show areas of fibrosis on April 24, 2021.

**Figure 2:**
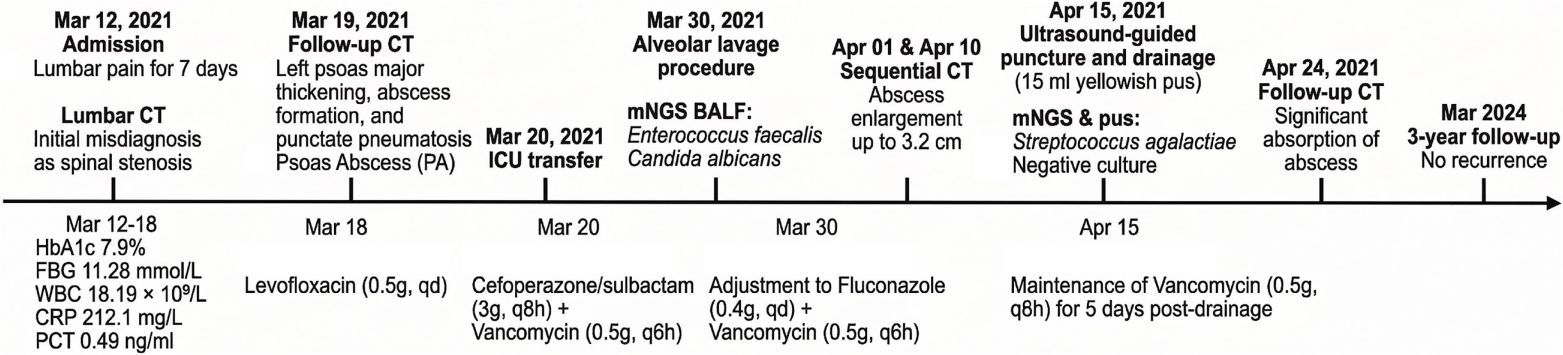
The clinical timeline of Case 1.

The patient is a 77-year-old woman who presented to the hospital on March 12, 2021, with numbness and pain in the right lower limb for more than 2 years, as well as lower back pain for the past 7 days. Seven days prior to admission, she experienced severe lower back pain, which was aggravated by positional changes and alleviated by lying down. Physical examination revealed a body temperature of 36.7 °C, interspinous step formation at the 4–5 lumbar spine level, and tenderness in the left buttock. A CT scan of the lumbar spine was initially interpreted as lumbar intervertebral disc herniation with spinal stenosis, with no abnormalities noted in the psoas major muscle. Laboratory tests indicated a glycated hemoglobin level of 7.9% and fasting blood glucose of 11.28 mmol/L, leading to a diagnosis of type 2 diabetes mellitus for the first time, for which insulin treatment was initiated. On March 18, leukocyte count was 18.19 × 10^9^/L, C-reactive protein was 212.1 mg/L, and procalcitonin was 0.49 ng/mL. Additionally, blood cultures were performed and returned negative, and relevant screening tests successfully excluded the possibility of a tuberculous abscess. Thus, levofloxacin (0.5 g, qd) was administered. A CT scan on March 19 revealed thickening of the left psoas major muscle, abscess formation, and punctate pneumatosis (Figure 1A), leading to a diagnosis of PA; bilateral pulmonary opacities suggestive of pneumonia, with bilateral pleural effusion. The patient was subsequently transferred to the ICU for treatment with cefoperazone/sulbactam (3 g, q8h) combined with vancomycin (0.5 g, q6h). On March 30, mNGS detected *Enterococcus faecalis* and *Candida albicans* in the alveolar lavage fluid, prompting an adjustment in treatment to fluconazole (0.4 g, qd) combined with vancomycin (0.5 g, q6h). However, a CT scan on April 1 indicated an enlargement of the abscess (Figure 1B). The abscess continued to increase in size, as confirmed by a CT scan on April 10 (Figure 1C). On April 15, 15 mL of yellowish pus was extracted via ultrasound-guided puncture and drainage, and mNGS identified *Streptococcus agalactiae*, while conventional cultures were negative. Following 5 days of vancomycin treatment (0.5 g, q6h), the abscess showed significant absorption (Figure 1D), and there was no recurrence over a follow-up period of 3 years. The total duration of antibiotic therapy for this patient was 38 days (Figure 2).

### Case 2

**Figure 3:**
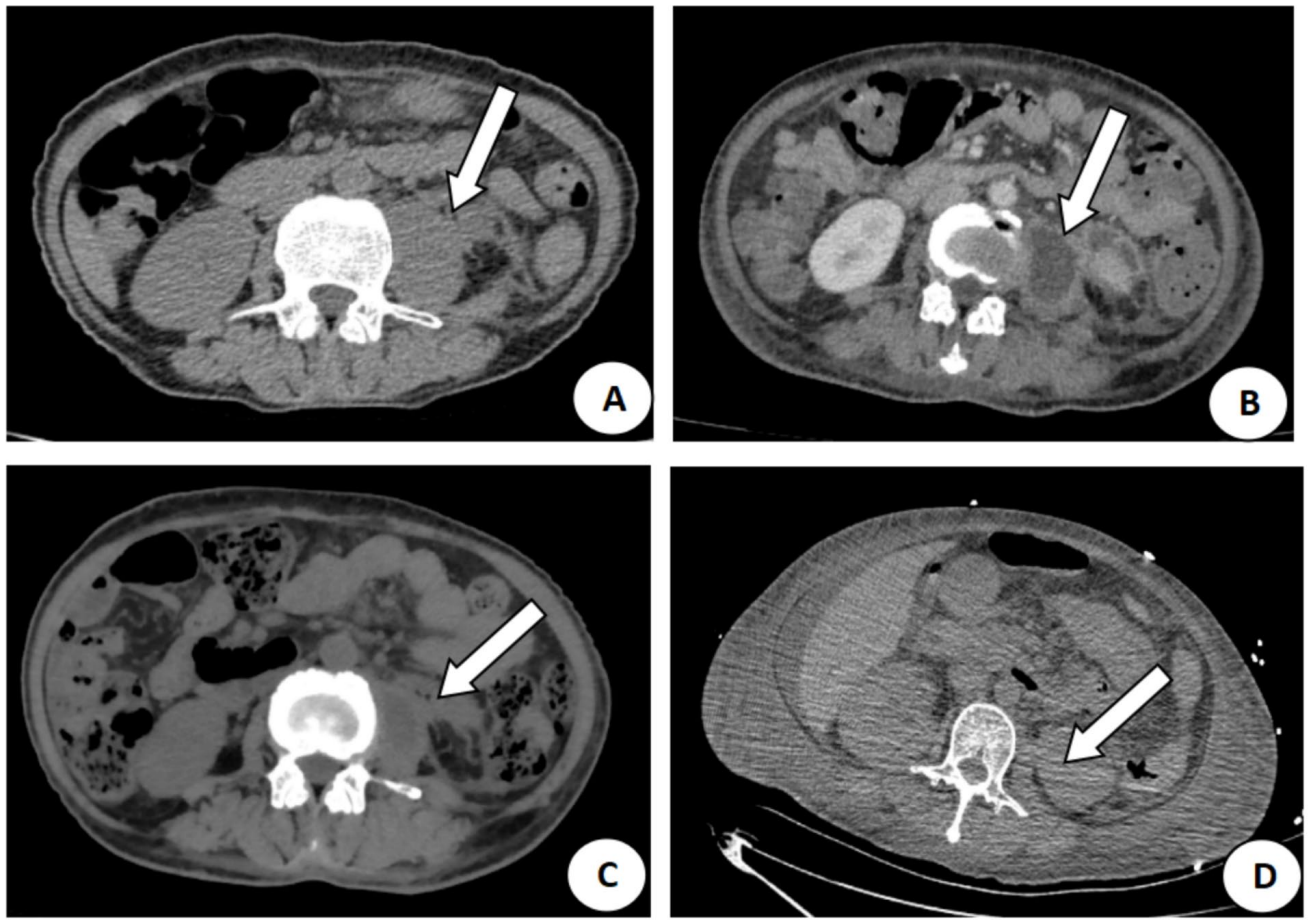
CT images of the lumbar major muscle of the patient in Case 2. **(A)** Swelling of the left lumbar major muscle, arrows show the extent of inflammation on June 30, 2021; **(B)** Abscess formation with a diameter of about 2.8 cm on July 9, 2021; **(C)** Reduction of the abscess to 1.2 cm, arrows show the residual pus cavity on July 23, 2021; **(D)** Complete resorption of the abscess, and arrows show the normal psoas major muscle structure on January 26, 2024.

**Figure 4:**
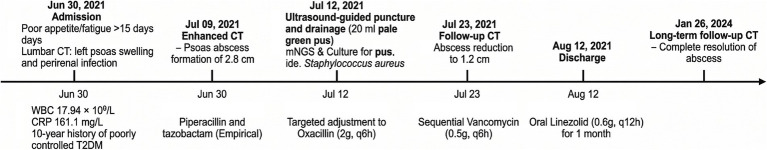
The clinical timeline of Case 2.

The patient was a 56-year-old woman who presented to the hospital on June 30, 2021, with a complaint of poor appetite and fatigue lasting over half a month, which was exacerbated by dizziness for 1 day. She had a 10-year history of diabetes mellitus with poor glycemic control and was admitted due to a urinary tract infection and left perirenal infection. Upon examination, percussion pain was noted in the left renal region, and blood tests revealed leukocytes at 17.94 × 10^9^/L and a C-reactive protein (CRP) level of 161.1 mg/L. Multiple blood cultures yielded negative results, and a tuberculous infection was carefully excluded through specific laboratory testing. A CT scan indicated swelling of the left lumbar psoas muscle and left perirenal infection (Figure 3A). The patient was treated with piperacillin and tazobactam, and a CT enhancement on July 9 suggested the formation of an abscess in the lumbar psoas muscle (Figure 3B). On July 12, ultrasound-guided puncture and drainage were performed, resulting in the extraction of 20 mL of pale green pus. Bacterial culture and mNGS identified *Staphylococcus aureus*, prompting a modification of treatment to oxacillin (2 g, q6h). Follow-up CT on July 23 showed a reduction in the size of the abscess (Figure 3C), leading to a change in treatment to sequential vancomycin (0.5 g, q6h). On August 12, the patient was discharged and continued to take linezolid (0.6 g, q12h) for 1 month. The patient was monitored until January 2024, when a follow-up CT indicated the complete resolution of the abscess (Figure 3D). The total duration of antibiotic therapy for this patient was 36 days (Figure 4).

## Discussion

The clinical manifestations of PA are diverse and often nonspecific, allowing categorization into primary and secondary forms (5). In this study, both patients presented with low back pain as the initial symptom, notably without accompanying fever or claudication. This observation suggests that diabetic patients may obscure early symptoms due to factors such as peripheral neuropathy, aligning with findings reported in the literature (6, 7). Therefore, low back pain in diabetic patients warrants heightened vigilance for potential complications associated with PA.

CT and MRI are widely utilized imaging modalities for diagnosing PA, as they effectively delineate the abscess’s location, size, and involvement of adjacent structures (8). In Case 1, the initial CT scan did not reveal PA, which may be attributed to atypical abscess formation in the early phase.

The treatment of PA necessitates adequate drainage alongside precise anti-infection measures (9). Ultrasound-guided percutaneous puncture drainage is noted for its low trauma and high safety profile (10). Although conventional diagnostic methods such as culture, are widely used in infectious diseases, they are limited by long processing times, narrow detection scopes, and poor capability for identifying unknown pathogens (11). mNGS, as a non-targeted, high-throughput detection technology, demonstrated significant advantages in diagnosing infection and identifying causative organisms compared with conventional culture (12, 13). In our study, mNGS successfully detected *S. agalactiae* despite negative conventional culture results and provided comprehensive insights into pathogen abundance, thereby confirming its efficacy in identifying low-load or fastidious bacteria in PA. These findings suggest that mNGS may help to improve the detection of pathogens, guide changes in treatment strategies, and is an effective complement to conventional culture. In a case of pelvic and PA caused by *M. pneumoniae*, which was successfully identified through mNGS. It also detected the presence of the A2063G mutation in the 23S rRNA gene, a key molecular mechanism conferring high-level resistance to macrolides (14). This finding further highlights an additional advantage of mNGS: its capacity to elucidate the molecular basis for the failure of empirical antibiotic therapy, thereby informing more precise clinical decision-making. However, it is limited by cost, infrastructure constraints, the computing power required for analyses of large data sets and drug sensitivity results (15, 16). Additionally, the patient in Case 1 experienced abscess progression due to inadequate empirical coverage before drainage, underscoring the importance of early intervention.

Elevated levels of inflammatory markers promote the development of T2DM and increase the risk of hyperglycemic crises in T2DM patients (17, 18). T2DM patients under poorly controlled conditions could develop rare inflammatory diseases, such as spontaneous discitis, pyogenic PA, spinal epidural abscess and bacterial meningitis (7). Therefore, glycemic control is crucial for effective treatment. HbA1c reflects poor glycemic control, which is closely associated with microvascular complications, delayed wound healing, and increased susceptibility to infections (19). Both cases demonstrated improved prognosis with intensive insulin therapy.

A study reported a patient diagnosed with a coinfection of perinephric and PA caused by *Lactobacillus johnsonii* and *T. vaginalis*, where the etiology was rapidly and accurately identified through mNGS (20). In our study, initial empirical treatment primarily included broad-spectrum antibiotics based on penicillin, and this class of antibiotics was also commonly used for *S. agalactiae* and *S. aureus* detected by mNGS; however, the therapeutic effect was limited. Therefore, subsequent antibiotic escalation to vancomycin was implemented based on the patients’ clinical response and mNGS results, which demonstrated significant clinical improvement and resolution of PA. These findings further highlight the role of mNGS as a critical tool for pathogen identification, optimization of antibiotic selection, and reduction of treatment duration. It is worth noting that while mNGS offers unparalleled advantages in rapid pathogen screening, its current technical framework typically does not provide phenotypic drug susceptibility results. Consequently, the ultimate selection of antibiotics should not rely solely on sequencing data but must be a synthesis of mNGS findings and the patient’s real-time clinical response, ensuring a more holistic and precise therapeutic approach. This report highlights that patients with diabetes mellitus combined with PA are more specific and clinically insidious, and indicates that mNGS may help to improve pathogen detection, guide adjustments in antibiotic treatment strategies, optimize treatment duration, and serve as an effective complement to conventional culture. Antibiotic resistance in patients should also be tested in future research. In addition, treatment of PA requires validation in larger studies.

## Data Availability

The original contributions presented in the study are included in the article/supplementary material, further inquiries can be directed to the corresponding author.

## References

[1] Xu C Zhou Z Wang S Ren W Yang X Chen H . Psoas abscess: an uncommon disorder. Postgrad Med J. (2024) 100:482–7. doi: 10.1093/postmj/qgad110, 38366872

[2] Rajbhandari SM Wilson RM. Unusual infections in diabetes. Diabetes Res Clin Pract. (1998) 39:123–8. doi: 10.1016/s0168-8227(97)00125-3, 9597382

[3] Kao PF Tsui KH Leu HS Tsai MF Tzen KY. Diagnosis and treatment of pyogenic psoas abscess in diabetic patients: usefulness of computed tomography and gallium-67 scanning. Urology. (2001) 57:246–51. doi: 10.1016/s0090-4295(00)00923-7, 11182330

[4] Li N Cai Q Miao Q Song Z Fang Y Hu B. High-throughput metagenomics for identification of pathogens in the clinical settings. Small Methods. (2021) 5:2000792. doi: 10.1002/smtd.202000792, 33614906 PMC7883231

[5] Agrawal SN Dwivedi AJ Khan M. Primary psoas abscess. Dig Dis Sci. (2002) 47:2103–5. doi: 10.1023/A:1019693400742 12353862

[6] Lansdown AJ Downing A Roberts AW Martin D. Psoas abscess formation in suboptimally controlled diabetes mellitus. Case Rep Med. (2011) 2011:249325. doi: 10.1155/2011/249325, 21811508 PMC3147154

[7] Horiya M Anno T Kawada M Yamada H Takahashi K Takenouchi H . Pyogenic psoas abscess on the dorsal side, and bacterial meningitis and spinal epidural abscess on the ventral side, both of which were induced by spontaneous discitis in a patient with diabetes mellitus: a case report. J Diabetes Investig. (2021) 12:1301–5. doi: 10.1111/jdi.13461, 33179391 PMC8264412

[8] Al-Khafaji MQ Al-Smadi MW Al-Khafaji MQ Aslan S Al-Khafaji YQ Bagossy-Blás P . Evaluating imaging techniques for diagnosing and drainage guidance of psoas muscle abscess: a systematic review. J Clin Med. (2024) 13:3199. doi: 10.3390/jcm13113199, 38892910 PMC11173313

[9] Yacoub WN Sohn HJ Chan S Petrosyan M Vermaire HM Kelso RL . Psoas abscess rarely requires surgical intervention. Am J Surg. (2008) 196:223–7. doi: 10.1016/j.amjsurg.2007.07.032, 18466865

[10] Kuhelj D Langel C. Image-guided percutaneous drainage of abdominal abscesses in pediatric patients. Children (Basel). (2024) 11:290. doi: 10.3390/children11030290, 38539325 PMC10969118

[11] Peng X Zhang L. Advances and challenges in the application of metagenomic sequencing for the diagnosis and treatment of infectious diseases: from pathogen spectrum identification to personalized antimicrobial strategies. Diagn Microbiol Infect Dis. (2026) 115:117321. doi: 10.1016/j.diagmicrobio.2026.117321, 41764831

[12] Yang A Chen C Hu Y Zheng G Chen P Xie Z . Application of metagenomic next-generation sequencing (mNGS) using Bronchoalveolar lavage fluid (BALF) in diagnosing pneumonia of children. Microbiol Spectrum. (2022) 10:e0148822. doi: 10.1128/spectrum.01488-22, 36169415 PMC9603332

[13] Jia K Huang S Shen C Li H Zhang Z Wang L . Enhancing urinary tract infection diagnosis for negative culture patients with metagenomic next-generation sequencing (mNGS). Front Cell Infect Microbiol. (2023) 13:1119020. doi: 10.3389/fcimb.2023.1119020, 36936777 PMC10020507

[14] Yu Y Che L Sun L Wang S Du N. A rare case of disseminated *Mycoplasma pneumoniae* infection spreading from a pelvic lesion to a psoas muscle abscess. Int J Infect Dis. (2026):108420. doi: 10.1016/j.ijid.2026.108420 41579934

[15] La Via L Ferlito S Di Modica MS Marino A Nunnari G Cacopardo B . The global impact of sepsis: epidemiology, recognition, management, and health system challenges. Epidemiologia. (2026) 7:20. doi: 10.3390/epidemiologia7010020, 41718052 PMC12921907

[16] Kapoor V Sanchez-Vicente S Donovan W Park J Nagapurkar A Gokden A . Adaptation of custom capture sequencing panels to the Oxford Nanopore MinION platform. Mol Biol Rep. (2026) 53:448. doi: 10.1007/s11033-026-11589-1, 41774281 PMC12956999

[17] Yang X Tao S Peng J Zhao J Li S Wu N . High-sensitivity C-reactive protein and risk of type 2 diabetes: a nationwide cohort study and updated meta-analysis. Diabetes Metab Res Rev. (2021) 37:e3446. doi: 10.1002/dmrr.3446, 33686799

[18] Tao LC Shu H Wang Y Hou Q Li JJ Huang XL . Inflammatory biomarkers predict higher risk of hyperglycemic crises but not outcomes in diabetic patients with COVID-19. Front Endocrinol (Lausanne). (2024) 15:1287795. doi: 10.3389/fendo.2024.1287795, 38455656 PMC10919215

[19] Hu Y Zhang Y Lin P Hu X Zhu Y Yan P . Interpretable machine learning model for predicting 1-year unplanned readmissions in ischemic stroke patients with diabetes: a synergistic view of inflammation and metabolism. Clin Interv Aging. (2025) 20:2163–75. doi: 10.2147/CIA.S544949, 41334071 PMC12667393

[20] Quan M Zhang X Chen C Feng Y Lv X Wang X . Perinephritic and psoas abscess by an unusual coinfection with *trichomonas vaginalis* and *Lactobacillus johnsonii*. Diagn Microbiol Infect Dis. (2026) 114:117276. doi: 10.1016/j.diagmicrobio.2026.117276, 41576635

